# Construction and Validation of a Compassionate Nursing Care Scale from the Perspective of the Patient-Family Caregiver Dyad

**DOI:** 10.17533/udea.iee.v42n3e12

**Published:** 2024-10-25

**Authors:** Edwin Darío Archila-Hernández, Doris Helena Torres-Acosta, Sandra Patricia Pulido-Barragán, Olga Lucía Laverde-Contreras, Beatriz Sánchez-Herrera

**Affiliations:** 1 Nurse; Master’s in Basic Biomedical Sciences. Nurse, Hospital Universitario de la Samaritana. Nursing Doctoral Student, Universidad de La Sabana. Email: enfermeria.jefe10@hus.org.co. https://orcid.org/0000-0002-8633-8031 Universidad de la Sabana Universidad de La Sabana Colombia enfermeria.jefe10@hus.org.co; 2 Nurse; master’s in nursing. Nursing Deputy Director. Hospital Universitario de la Samaritana. Email: enfermeria.subdirect@hus.org.co. https://orcid.org/0009-0005-6550-4327 Hospital Universitario de la Samaritana Hospital Universitario de la Samaritana Colombia enfermeria.subdirect@hus.org.co; 3 Nurse; Master’s in Nursing, Specialist in Health Audit, Specialist in Health Administration. Project leader. Hospital Universitario de la Samaritana. Email: enfermeria.liderdp@hus.org.co. https://orcid.org/0000-0002-7512-0010 Hospital Universitario de la Samaritana Hospital Universitario de la Samaritana Colombia enfermeria.liderdp@hus.org.co; 4 Nurse; Master’s in Nursing. Professor, Universidad de La Sabana. Email: lulagreen28@gmail.com. https://orcid.org/0000-0002-0851-0772 Universidad de la Sabana Universidad de La Sabana Colombia lulagreen28@gmail.com; 5 Nurse; Master of Science in Nursing, Gerontological Nurse Practitioner. Professor, Universidad de La Sabana. Email: publifer@unisabana.edu.co. Corresponding author. https://orcid.org/0000-0002-8029-7187 Universidad de la Sabana Universidad de La Sabana Colombia publifer@unisabana.edu.co

**Keywords:** nursing methodology research, empathy, nursing care, quality of health care, nursing theory., metodológica en enfermería, compasión, atención de enfermería, calidad de la atención de salud, teoría de enfermería., investigação metodológica em enfermagem, compaixão, cuidados de enfermagem, qualidade dos cuidados de saúde, teoria de enfermagem.

## Abstract

**Objective.:**

This work sought to determine the composition, comprehensibility, face validity, and content validity of the Compassionate Care Scale “HUS-CC”, to assess the perception of the patient-family caregiver dyad on the compassionate nursing care they receive in hospital services.

**Methods.:**

Methodological research conducted in a hospital network with services at different levels of complexity in Colombia. To evaluate the comprehensibility of the HUS-CC, 204 individuals participated. The face and content validity were valued by 17 experts and ratified with 213 dyads. An exploratory factor analysis with varimax rotation was conducted and reliability was calculated with Cronbach’s alpha.

**Results.:**

The scale was clear and understandable. Its face and content validity index were 0.77; the Fleiss Kappa index was 0.59 and Aiken’s V was 0.9; with Cronbach’s alpha of 0.84. The HUS-CC has 16 items grouped into three dimensions: warm treatment, inclusive attitude, and supportive behavior.

**Conclusion.:**

The HUS-CC scale proved valid to evaluate the perception of patient-family caregiver dyads about compassionate nursing care in hospital services.

## Resumen

**Objetivo.:**

Determinar la composición, comprensibilidad, validez facial y validez de contenido de la Escala de Cuidado Compasivo “HUS-CC”, para evaluar la percepción de la díada paciente-cuidador familiar sobre el cuidado compasivo de enfermería que reciben en servicios hospitalarios.

**Métodos.:**

Investigación Metodológica desarrollada en una red hospitalaria con servicios de diferente nivel de complejidad en Colombia. Para valorar la comprensibilidad de la HUS-CC, participaron 204 personas. La validez facial y de contenido fue valorada con 17 expertos y ratificada con 213 diadas. Se realizó un análisis factorial exploratorio con rotación varimax y se calculó la fiabilidad con el alfa de Cronbach.

**Resultados.:**

La escala fue clara y comprensible. Su índice de validez facial y de contenido fue de 0,77; el índice Kappa de Fleiss de 0,59 y el V de Aiken de 0,9. El alfa de Cronbach fue de 0,84. La HUS-CC tiene 16 ítems agrupados en tres dimensiones: trato cálido; actitud incluyente y conducta de apoyo.

**Conclusión.:**

La escala HUS -CC demostró ser válida para valorar la percepción de las díadas paciente-cuidador familiar sobre el cuidado compasivo de enfermería en servicios hospitalarios.

## Resumo

**Objetivo.:**

Determinar a composição, compreensibilidade, validade facial e validade de conteúdo da Escala de Cuidado Compassivo “HUS-CC”, para avaliar a percepção da díade paciente - cuidador familiar sobre o cuidado de enfermagem compassivo que recebe nos serviços hospitalares.

**Métodos.:**

Pesquisa metodológica desenvolvida em uma rede hospitalar com serviços de diferentes níveis de complexidade na Colômbia. Para avaliar a compreensibilidade do HUS-CC participaram 204 pessoas. A validade facial e de conteúdo foi avaliada com 17 especialistas e ratificada com 213 díades. Foi realizada análise fatorial exploratória com rotação Varimax e a confiabilidade foi calculada com alfa de Cronbach.

**Resultados.:**

A escala era clara e compreensível. Dele o índice de validade facial e de conteúdo foi de 0.77; o índice Fleiss Kappa de 0.59 e o Aiken V de 0.9. O alfa de Cronbach foi de 0.84. O HUS -CC possui 16 itens agrupados em três dimensões: tratamento caloroso; atitude inclusiva e comportamento de apoio.

**Conclusão.:**

A escala HUS-CC mostrou-se válida para avaliar a percepção das díades paciente-cuidador familiar sobre o cuidado compassivo de enfermagem em serviços hospitalares.

## Introduction

Compassionate care is that which is provided consciously and from a bond with a suffering person who is sought to be relieved; this care requires a reflection and action process by the caregiver.^1^ It is also a way of expressing the transcendence of the human condition.^2^ In the health field, caring compassionately has permitted to improve interpersonal relations generating positive impact between health providers and its users.^3^

For nursing, providing compassionate care implies that its service has attributes, like wisdom, humanity, and empathy and that these are expressed in front of people who are vulnerable or suffering.^4^ This type of care requires for nurses, in addition to knowledge and skills, to maintain warm and friendly communication through actions, such as a smile, holding hands, looking and actively listening, an appropriate tone of voice, a good sense of humor, or any expression on their part that takes into account the culture and respect for patients and their family caregivers.^5^ To identify the needs of the people in their care, understand their health beliefs and facilitate the contribution that they themselves can make to promote and recover their health and well-being, it is important for nurses to involve patients and their family caregivers, to be available when their services are required, offer timely care, supervise the evolution and development of tasks under their care, and ensure that the care and context in which it is provided respond to what its users require and expect.^6^ In addition, training in compassionate care is associated with better nursing performance, which can support the recovery, satisfaction, and experience of patients and their family caregivers during their time in the health institution.^7^ In that sense, knowing the experience of patients and their family caregivers is important for the nursing staff because it allows them to assess the care during hospitalization and, thus, carry out activities that can improve the quality and safety of care, including greater cooperation, agreement, and decisions about care plans, along with their implementation and evaluation.^8^

Several tools have been developed to evaluate compassionate nursing care, these include the Compassion Competence Scale - an self-report instrument on compassionate nursing competence that reports a Cronbach’s α of 0.91, has 17 items, and includes the dimensions of communication, sensitivity, and knowledge.^9^ The Compassion Scale that values the perception of patients about the comprehensive concern for their suffering and inclination to help them; it reports a Cronbach’s α of 0.94 and includes the characteristics: cold/warm, unpleasant/pleasant, distant/compassionate, insensitive/sensitive, and indifferent/affectionate.^10^ The Compassionate Care Assessment tool that evaluates holistically in-hospital nursing care, with Cronbach’s α of 0.81 and 28 items grouped into the dimensions of significant connection, patient’s expectations, attributes of care, and professional capacity.^11^ The Schwartz Center Compassionate Care Scale, which measures patients’ perspectives about compassionate care by the health staff during hospitalization, is one-dimensional, has 12 items, reports Cronbach’s α of 0.76 - 0.95, and includes empathic concern and care and tenderness for those who suffer.^12^ The Sinclair Compassion Questionnaire that measures patients’ reports about compassionate care; it is one-dimensional with 15 items and Cronbach’s α of 0.96. Its authors consider it the Gold Standard for this measurement.^13^ Also, complementing the previous compassionate care measurement tools, there is the Compassion Practice Scale that, unlike the previous scales, seeks to evaluate to what extent a hospital fosters compassionate care in its employees; it is one-dimensional and includes five items and a Cronbach’s α of 0.82.^14^

Despite progress in measurements of compassionate care applicable to the practice by nursing and other professionals, No clinical tools were found in the world literature that consider the perception of compassionate care by patient-family caregiver dyad as a subject of care. Also, no reports exist of compassionate care tools that have been validated in Latin America. In that sense, this study sought to determine the composition, comprehensibility, and face and content validity of the HUS-CC tool that evaluates the perception of patient-family caregiver dyads about the compassionate nursing care they receive in Colombian hospital services.

## Methods

This was a nursing methodological research carried out in a teaching-care alliance, which sought the construction and validation of an instrument to measure compassionate nursing care from the perspective of patient-family caregiver dyads. It was developed by phases, thus: 

Phase 1. Identification of the need and development of the tool to measure the perception by the patient-family caregiver dyad on the level of compassionate nursing care. The Nursing Professional Practice model that guides the Hospital Universitario de la Samaritana (HUS) network, where this study took place, seeks leadership by nursing in compassionate and safe care. The subject of care are the patient-family caregiver dyads. According with its goals, the level of compassionate care perceived by the dyads must be measured to maintain or improve it continuously. To develop the compassionate care scale, a literature review was conducted with support from the EUREKA metasearch engine that includes 35 databases, like PubMed and SciELO and in CINAHL with the following search formula: [(Patient OR Caregiver OR Dyad) AND (Compassionate Care OR Compassion OR Compassionate Nursing) AND (Assessment OR Assessment Tool OR Evaluation)]. Dates, languages, or geographic sites were not limited. Of the 442 studies reported, 110 were selected from the title and abstract to be fully reviewed. Of those, 47 articles were considered due to their contribution to this study. Based on the identified need and the literature review, the research group developed a proposal of a scale to measure the perception of the patient-family caregiver dyad on the level of compassionate care provided by nursing.

Phase 2. Assessment of the scale’s comprehensibility level. The level of clarity of the proposed scale was revised by 204 people, patients or family caregivers, with different degree of schooling, socioeconomic status, age, and gender. Each person was asked through a questionnaire in Google Forms if each of the items on the proposed scale was understandable or not, asking them to make the observations they deemed necessary in front of each question. The degree of comprehensibility of the items was determined by calculating the percentages obtained, where a percentage > 85% was considered high comprehensibility.^15^ If the item did not fulfill that criterion, it’s wording was revised and adjusted. 

Phase 3. Face and content validity of the scale. The scale was validated through the judgment of 17 national or foreign experts who fulfilled the following criteria: having command of Spanish, being professionals with a graduate degree in any field of health or social areas related to care, and having more than five years of experience working with care issues. Each expert received a validation form together with the proposed scale and independently evaluated such, keeping in mind four criteria: clarity, relevance, coherence, and sufficiency of each item and dimension. In each case, they were asked to score in a Likert scale if the criterion was not met, 1; if there was a low level of compliance, 2; if the level of compliance was moderate, 3; and if there was full compliance, 4. Thereafter, these results were incorporated onto an Excel spreadsheet and analyzed under Lawshe parameters modified by Tristán,^15^ weighting their content validity ratio (CVR) per item and the content validity index (CVI) for the scale as a whole. According with these parameters, agreement among experts with values > 0.582 was accepted.^16^ Then, Aiken’s V (AV) index was calculated to quantify item relevance with respect to the domains, accepting levels > 0.75 as valid.^17^ Lastly, the Fleiss Kappa index was calculated to assess reliability among evaluators, whose level of agreement was valued as follows: 0.00 poor, from 0.1 to 0.20 slight, from 0.21 to 0.40 acceptable, from 0.41 to 0.60 moderate, from 0.61 to 0.80 considerable, and from 0.81 to 1.0 almost perfect.^15^

Phase 4. Construct validity and reliability of the scale. Once the scale was adjusted and validated by the experts, it was applied to a group of 213 dyads who were receiving services in the hospital network and who accepted to participate by responding voluntarily. The results were entered into an Excel database and, subsequently, validated with the Jamovi Tool to perform an exploratory factor analysis after measuring the Kaiser Meyer Olkin (KMO) and Bartlett's sphericity assumptions.^18^ The maximum likelihood with varimax rotation was used as the extraction method. The reliability of the scale was evaluated by calculating Cronbach's alpha, which was interpreted as follows: from 0.01 to 0.20 very low, from 0.21 to 0.40 low, from 0.41 to 0.60 moderate, from 0.61 to 0.80 high, and from 0.81 to 1.00 very high.^19^

Ethical aspects. The study received informed consent from the participants and was endorsed by the institutions involved after reviewing ethical and environmental aspects (Act No. 003140319).

## Results

Phase 1. Identification of the need and development of the scale to measure the perception of the patient-family caregiver dyad about the level of compassionate nursing care. From the literature review, it was possible to identify the characteristics and dimensions that reflect compassionate care in the nursing practice and which were present in the different evaluation tools reported. With this input, the preliminary version was generated of the Compassionate Care Scale denominated HUS-CC, for the acronym of the Hospital Network in which it was developed. This version included 16 items distributed into five dimensions: prioritizes the person with three items, treats warmly with three items, educates for care with four items, models care with three items, and facilitates care with three items. 

Phase 2. Assessment of the scale’s comprehensibility level. The participants’ characteristics reflected their heterogeneity. Of this group, 79% were women and 21% men. Their educational level was of primary for 7.4%, high school for 11.3%, technical or technological formation for 47.1%, professional formation for 29.9%, and graduate formation for 4.4%. Their socioeconomic level according to the housing strata was low (strata 1 to 3) in 96.6% and high (strata 4 to 6) in 3.4%. The comprehensibility tests of the HUS-CC scale showed results between 87.7 % and 100%, thus, no semantic adjustments were required in the items proposed. 

Phase 3. Face and content validity of the scale. From the concept by the experts about the scale, a CVR was found between 0.65 and 0.92 and CVI of 0.77 for the total test. Aiken’s V index was 0.90, with values fluctuating between 0.81 and 0.98 (Table 1). 

**Table 1:** Content validity analysis of the HUS-CC scale conducted by the 17 experts

Item	Analysis according to Lawshe parameters				Analysis according to Aiken parameters			
	Clarity	Relevance	Coherence	Suffi-ciency	CVR	Clarity	Relevance	Coherence	Suffi-ciency	Sub-total
1	0.82	0.94	0.88	0.68	0.83	0.92	0.96	0.94	0.89	0.93
2	0.47	0.88	0.82		0.71	0.76	0.96	0.92		0.89
3	0.52	0.82	0.82		0.71	0.76	0.92	0.92		0.87
4	0.70	0.94	0.88	0.76	0.82	0.88	0.96	0.94	0.92	0.93
5	0.94	1	1		0.92	0.98	1	1		0.98
6	0.94	1	1		0.92	0.98	1	1		0.98
7	0.82	0.70	0.70	0.52	0.69	0.88	0.74	0.80	0.81	0.81
8	0.88	0.82	0.82		0.76	0.94	0.82	0.88		0.87
9	0.58	0.82	0.94		0.72	0.80	0.86	0.94		0.84
10	0.88	0.88	0.94	0.63	0.83	0.96	0.96	0.98	0.86	0.94
11	0.70	0.88	0.88		0.77	0.9	0.90	0.92		0.90
12	0.64	0.94	0.94		0.79	0.88	0.96	0.92		0.90
13	0.82	0.88	0.94		0.82	0.92	0.94	0.98		0.93
14	0.52	0.7	0.76	0.60	0.65	0.80	0.88	0.90	0.87	0.86
15	0.58	0.88	0.82		0.72	0.84	0.96	0.94		0.91
16	0.64	0.88	0.82		0.74	0.88	0.96	0.94		0.91
n = 17	CVI 0.77				AV 0.90

Table 2 shows the strength of agreement among evaluators for the HUS-CC scale, which was 0.59 for the total. The dimensions of Treats warmly and Educates for care obtained the highest values with 0.79 and 0.63, respectively. 

**Table 2:** Strength of agreement among evaluators for the HUS-CC scale

Dimensions	Attributes			Fleiss Kappa Coefficient		Strength of agreement
	Clarity	Relevance	Coherence	Sufficiency		
Prioritizes the person	0.34	0.72	0.62	0.45	0.53	Moderate
Treats warmly	0.73	0.95	0.91	0.56	0.79	Considerable
Educates for care	0.57	0.75	0.82	0.39	0.63	Considerable
Models care	0.55	0.54	0.62	0.32	0.51	Moderate
Facilitates care	0.35	0.62	0.58	0.42	0.49	Moderate
Total	0.51	0.72	0.71	0.43	0.59	Moderate

Phase 4. Construct validity and scale reliability. The resulting version of the HUS-CC was applied to 213 patient-family caregiver dyads from the three institutions that make up the Hospital Network where the study was conducted. Kaiser’s exploratory factor analysis was 0.83. The correlation of the HUS-CC scale, evaluated with Bartlett’s sphericity test indicated significance < 0.001, which permitted rejecting the null hypothesis and proceeding to the factor analysis. Upon regrouping the items with the exploratory factor analysis, the final version was generated of the HUS-CC scale with 16 items distributed in three dimensions: 1) warm treatment, with eight items; 2) inclusive attitude, with four items; and 3) supportive behavior, with four items (Table 3). 

**Table 3:** Exploratory factor analysis of the HUS-CC scale

Category	Item	Factor		
		1	2	3
Warm treatment	Calls them by their name	0.553		
	Recognizes them as persons	0.544		
	Serves them with priority	0.335		
	Respects them	0.448		
	Listens to them attentively	0.343		
	Treats them kindly (warmth)	0.489		
	Is patient with them	0.426		
	Inspires confidence in them	0.718		
Inclusive attitude	Explains the procedures carried out		0.584	
	Involves them in care		0.678	
	Teaches them about care		0.789	
	Asks about what he/she has taught them		0.636	
Supportive behavior	Sets an example with his/her care behavior			0.498
	Accompanies them permanently			0.730
	Facilitates care when it is complex			0.554
Supports them during treatment and recovery			0.443

As overall instructions before starting the questionnaire, patients, caregivers, or family participants were told: "Please indicate your level of agreement with each of the following statements based on the care you have received from the institution's nursing staff. Indicate in each case if you totally agree, partially agree, disagree, or completely disagree with each of them”. Finally, the HUS-CC scale had a Cronbach’s alpha of 0.84, indicating its high reliability.

## Discussion

The HUS-CC scale, designed and validated, shows essential elements that characterize compassionate nursing care. Among them, those proposed by Burnell who states that compassion in care is the outcome of an authentic bond between a nurse and a patient that must reflect comprehension and sensitivity to the distressing reality and suffering of the recipient of care and generate actions that seek to alleviate such.^20^ Similarly, it reflects the characteristics proposed by Taylor *et al*., as care characterized by recognition, connection, and an altruistic impulse desire with humanistic responses and actions.^21^ It also takes into account the call by Llarde *et al*., for compassionate care to besides being the fundamental characteristic in the quality of nursing care or a distinctive seal of a theoretical model, it should be the result of a genuine connection during nursing care that must - in turn - focus on the patients that demand it, considering their perception.^22^ As evidenced, the HUS-CC scale includes the warm treatment, inclusive attitude, and supportive behavior by nurses during care.

The view of compassionate care from different perspectives including patients or their family caregivers, individually or as dyad, complements contributions like that made by Diaz *et al*., who evaluated compassionate care from the perspective of students as caregivers^23^ and by Papadopoulos *et al*., who assessed this care from the opinions by nurses from 15 countries.^24^ However, as indicated by these authors, putting compassionate nursing care into practice needs further research to explore not only different perspectives but also their differences in different cultures. 

Compassionate care in nursing, as reflected by the HUS-CC scale, is essentially comprised of soft skills that must be measured to evidence and improve them, as noted in the works by Lee and Seomun;^9^ Fogarty *et al*.;^10^ Burnell *et al*.;^11^ Lown *et al*.,:^12^ and Sinclair *et al*.^13^ The evaluation of the attributes of compassionate care achieved through this scale, responds to the proposals by Llarde *et al*., who indicate that although the concept of compassionate care is central in the nursing practice, the measurement or improvement of this type of care is not evident in this practice.^22^

To end, this development responds to the proposal by McClelland and Vogus,^14^ contributing to the measurement of compassionate care in different scenarios and levels of care. However, it is a limitation of the present study that the scale does not allow measuring compassionate care from the nurse’s perspective as an indispensable part in the construction of the care bond. Hence, it is necessary to continue studying to design a scale that can be used with nursing to assess its perspective about its capacity to offer compassionate care. Likewise, it will be necessary to also respond - as indicated by Colletti *et al*.,^25^ to the institution’s responsibilities, which as a university hospital it has in relation to training, with an analysis of the impact this scale or its adaptation may have on the development of human talent skills. 

The HUS-CC constructed through a teaching-nursing assistance alliance and designed to be applied to patients, family caregivers or the dyad, by a previously trained healthcare worker, permits visualizing, measuring, and improving the compassionate nursing care practice and could be useful to enhance formation in this field. This scale is constituted by three categories that include warm treatment, with eight items; inclusive attitude, with four items; and supportive behavior with four items. It is measured via a four-option Likert scale that includes totally agree, partially agree, partially disagree, and totally disagree. Its validation reflected that it is a clear and understandable scale, with adequate content and structure to be applied in Colombian population.
